# Reduced Bone and Body Mass in Young Male Rats Exposed to Lead

**DOI:** 10.1155/2014/571065

**Published:** 2014-03-30

**Authors:** Fellipe Augusto Tocchini de Figueiredo, Raquel Fernanda Gerlach, Márcia Andreia Mesquita Silva da Veiga, Flavio Venancio Nakadi, Junia Ramos, Erika Reiko Kawakita, Carolina de Souza Guerra, João Paulo Mardegan Issa

**Affiliations:** ^1^Faculdade de Odontologia de Ribeirao Preto, Universidade de Sao Paulo, Avenida do Cafe S/N, 14040-904 Ribeirao Preto, SP, Brazil; ^2^Faculdade de Filosofia Ciencias e Letras de Ribeirao Preto, Universidade de Sao Paulo, Avenida do Cafe S/N, 14040-904 Ribeirao Preto, SP, Brazil

## Abstract

The aim of this study was to see whether there would be differences in whole blood versus tibia lead concentrations over time in growing rats prenatally. Lead was given in the drinking water at 30 mg/L from the time the dams were pregnant until offspring was 28- or 60-day-old. Concentrations of lead were measured in whole blood and in tibia after 28 (28D) and 60 days (60D) in control (C) and in lead-exposed animals (Pb). Lead measurements were made by GF-AAS. There was no significant difference (*P* > 0.05) in the concentration of whole blood lead between Pb-28D (8.0 ± 1.1 **μ**g/dL) and Pb-60D (7.2 ± 0.89 **μ**g/dL), while both significantly varied (*P* < 0.01) from controls (0.2 **μ**g/dL). Bone lead concentrations significantly varied between the Pb-28D (8.02 ± 1.12 **μ**g/g) and the Pb-60D (43.3 ± 13.26 **μ**g/g) lead-exposed groups (*P* < 0.01), while those exposed groups were also significantly higher (P < 0.0001) than the 28D and 60D control groups (Pb < 1 **μ**g/g). The Pb-60D group showed a 25% decrease in tibia mass as compared to the respective control. The five times higher amount of lead found in the bone of older animals (Pb-60D versus Pb-28D), which reinforces the importance of using bone lead as an exposure biomarker.

## 1. Introduction

Lead is a highly toxic metal and has a wide distribution for its great usefulness. Several studies have linked health problems with high concentrations of industrial minerals, elements coming from contaminated rivers, and carelessness of the public service of water [1, 2]. According to a US study from 2003, data provided by the 1999-2000 NHANES study estimated that approximately 0.45 million children under 6 years of age showed whole blood lead levels exceeding or equal to 10 *μ*g/dL Pb, which were considered high blood lead levels for children [3]. Even though lead levels have decreased in many countries in the last decades, it is now known that even low lead levels, which do not cause symptoms of acute poisoning, are associated with cognitive and neurological disorders [4].

Bone is considered the best marker for lead exposure [5], but it cannot be used for lead determinations in humans yet. Nonetheless, in animals bone lead determinations are relatively easy to perform so far as sacrifice of animals is concerned. The determinations of lead in bone do have some analytical challenges, for instance, the lack of reference materials for lead in bone. On the other hand, bone lead determinations also have some advantages—even from an analytical point of view. For instance, when planning lead determinations by ion-coupled plasma mass spectrometry, the bone acid extract is more convenient than the analysis of whole blood.

We have recently exposed female rats to both lead and fluoride, before they got pregnant, and we have determined lead and fluoride in the female offspring when these animals were 81-day-old [6]. For that study, we compared lead in different mineralized tissues and lead in whole bone, and there was high consistency among measurements. When discussing those data, we have found only a few studies in the literature that showed the amount of lead stored in bone when animals were chronically exposed to low lead levels, in comparison to dozens of studies that determined lead in whole blood.

Since exposure to relatively low lead levels is known to have consequences in many physiological and psychological aspects for both humans and animals, we have wondered whether the lead determinations in whole blood and in bone would agree after a short exposure time (28 and 60 days with exposure starting at pregnancy) in young rats exposed to low lead levels. To our knowledge, the comparison of whole blood versus bone lead levels has not been made in animals exposed to relatively low lead levels, such as the 30 mg/L dose (given in the drinking water) used in this study.

Doing a literature search using the words “rat,” “lead” (which actually introduces many unrelated studies), and “bone,” the number of published studies that determined lead in whole blood and bone is not large, and those studies are presented in Table 1. In most of those studies, lead is provided in the drinking water, and the concentrations of lead given vary from 50 to 1000 mg/L.

To our knowledge, the comparison of whole blood versus bone lead levels has not been made in animals exposed to relatively low lead levels, such as the 30 mg/L dose used in this study. Therefore, this study aimed at determining lead in whole blood and in tibia bone of 28- and 60-day-old male rats, as well as assessing growth by measuring body weight and tibia weight at 60 days.

## 2. Materials and Methods

### 2.1. Animals

This study was approved by the Ethics Committee for Experimentation with Animals, University of São Paulo, Campus of Ribeirão Preto, Brazil, under protocol number 07.1.346.53.3 and complies with the international guidelines for the use of animals in experimentation. Forty-nine male Wistar rats were used, divided in the following groups: 28-day-old controls (C-28D, *n* = 10) and 28-day-old lead-exposed rats (Pb-28D, *n* = 10) and 60-day-old controls (C-60D, *n* = 12) and 60-day-old lead-exposed rats (Pb-60D, *n* = 17). Water was provided* ad libitum.* Animals exposed to lead received water containing lead since birth up to 28 or 60 days. Lead was provided in the drinking water at 30 mg/L of lead in the form of lead acetate (CH_3_COO(Pb)2.3H_2_O) per 1 liter of deionized water. At the end of the experiment, the animals were anesthetized by ketamine 100 mg/kg and xylazine 10 mg/kg intraperitoneally. One milliliter of whole blood was collected via cardiac puncture with a heparinized syringe, whose preparation is described below. The animals were then sacrificed by anesthetic overdose, and tibiae were collected. Tibiae were completely freed of soft tissue, maintained at 40°C for 48 hours, and weighed on an analytical balance. The level of significance for the differences accepted in this study was *P* < 0.05.

### 2.2. Equipment and Materials

Lead was determined by Graphite Furnace Atomic Absorption Spectrometry (GF-AAS) on AA600 model (Perkin Elmer, USA). The inert protective gas purge was argon with 99.999% of purity (White Martins, São Paulo, Brazil). All glassware and plastics used were properly cleaned with nitric acid to avoid contamination. For this study, the following limits of detection (LOD) and quantification (LOQ) were obtained: LOD: 0.4 mg/L and LOQ: 1.2 mg/L.

### 2.3. Whole Blood Sample Preparation

Blood was collected in plastic syringes that were previously decontaminated with nitric acid. Needles, syringe, and metal free falcon were rinsed with sodium heparin 5000 mg/ml, and, after collection preparation, the blood was collected (an average of 1 ml). Thereafter blood samples were stored at −20°C, being analyzed by GF-AAS.

### 2.4. Whole Blood Lead Determination

Lead was determined in whole blood following the method described by Parsons and Slavin [7], where the modifier comprises 0.2% NH_4_H_2_PO_4_, 0.5% Triton X-100, and 0.2% HNO_3_, which was used as the sample diluent. NIST 955c was used as a standard with known amount of lead after every 15 samples. Samples and standard, prepared as described above, were diluted 1 : 10 into 1 ml cups and placed in the sampler AS 800 (Perkin Elmer, USA). Twelve *μ*l aliquots were then automatically pipetted into the Zeeman-type graphite tube.

## 3. Results and Discussion

The lead concentrations found in whole blood are shown in Table 2, being 1.2 *μ*g/dL (±0.7) in the C-28D, 1.6 *μ*g/dL (±1.5) in the C-60D, 8.0 *μ*g/dL (±1.1) in the group Pb-28D, and 7.2 *μ*g/dL (±0.89) in the group Pb-60D, with significant differences when groups of animals exposed to lead were compared with the controls (*P* < 0.001), but with no significant differences found between the groups exposed to lead for 28 and 60 days.

**Figure 1:**
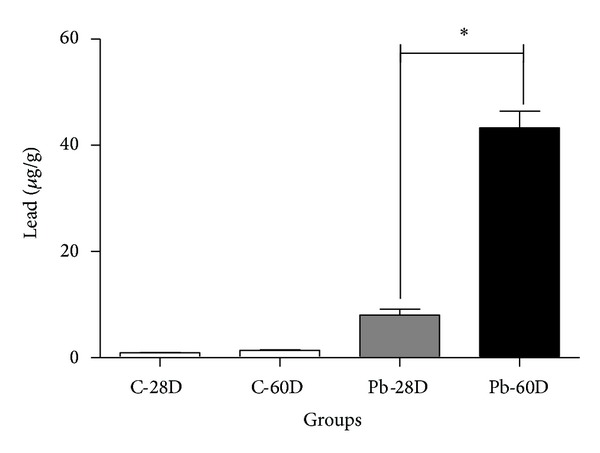
Lead in tibia bone after 28 and 60 days of exposure to lead. Lead concentrations in tibias of 28- and 60-day-old rats.

In contrast, the lead concentrations found in the bone samples (Figure 1) were 8.2 *μ*g/g (±1.1) in the Pb-28D group and 43.3 *μ*g/g (±13.2) in the Pb-60D (*P* < 0.0001), while the C-28D group showed 1.5 *μ*g (±1.1 *μ*g/g) and the C-60D group showed 2.3 *μ*g (±1.8 *μ*g/g) (the difference for those groups versus the respective lead-exposed groups was significant at *P* < 0.001 and *P* < 0.0001, resp.). The number of samples for those groups was C-28D (*n* = 10), C-60D (*n* = 9), Pb-28D (*n* = 12), and Pb-60D (*n* = 12).

**Figure 2:**
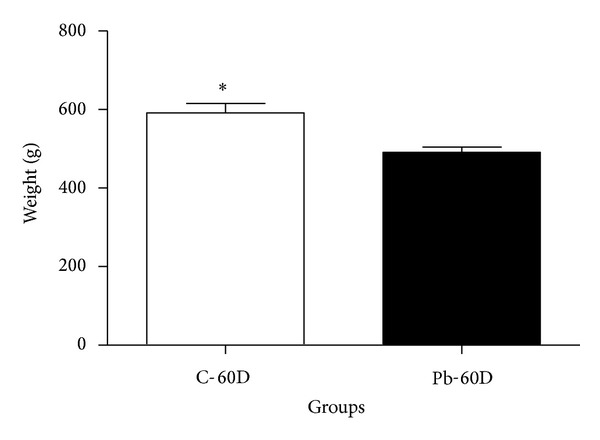
Body weight (g) of control and Pb-exposed rats after 60 days. Body weight at 60 days.

**Figure 3:**
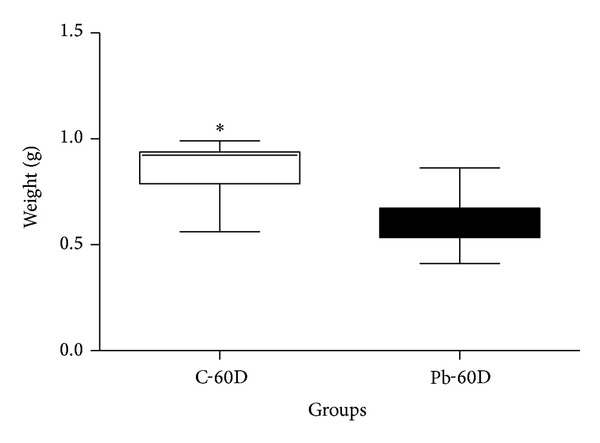
Weight (g) of tibia of control and Pb-exposed rat after 60 days. Tibia weight of rat aged 60 days.

In this study, we also compared whether this relatively low dose of lead had consequences for bone mass development and animal's weight gain (body weight).  Figure 2 shows body weight of 60-day-old animals in grams (g). Sample number was *n* = 12 in the 60-day control group (C-60D) and *n* = 16 in the Pb-60D (*n* = 16). Data distribution was normal. Unpaired Student's* t*-test shows a statistically significant difference (*P = 0.0006) between controls and lead-exposed animals. In Figure 3, tibia weight was expressed in grams (g), and sample number was *n* = 12 in the C-60D group, and *n* = 17 in the Pb-60D. Differences were statistically significant at *P* = 0.0004 (Mann-Whitney test).

A decrease in body weight (or body mass) of the Pb-60D animals was observed (*P* < 0.01 for comparison with age-matched controls and with the Pb-28D animals). The weight decrease was 17% of the C-60D animals' weight (Figure 2).

Tibia weight was also decreased in the Pb-60D group, a larger decrease (~25%) when the Pb-60D was compared with the age-matched control (*P* < 0.0004) (Figure 3). This suggests that the changes in bone biology are to some extent independent of the metabolic changes that affect body mass. Results of a decreased total body mass are in accordance with other studies on the effects of lead in rodents. The study of Conti et al. (2012) [8] showed a 10% decrease in body mass of lead-exposed animals in comparison to controls, but lead doses in that study were much higher, as described below.

Delayed growth with decreased body mass for age was also observed with doses of 50 mg/L and 250 mg/L of lead in the drinking water for 270 and 180 days, respectively [13]. The timing between 60 and 90 days has been described to be particularly important for lead incorporation, since this time window is characterized by rapid growth in rodents [14]. This is the probable reason why we only observed differences in total body mass and tibia mass in the Pb-60D group. Regarding the effect of lead on a decrease in bone mass, there have been already descriptions of lead leading to decreased bone mass, but with much higher doses. Female rats with 21 days of age were exposed to 1000 mg/L of lead acetate for 90 days, showing an average of 632.29 mg/g (±94.23) of lead in bone ash [8]. In another study, male rats were exposed to 500 mg/L of lead acetate during 12 weeks, reaching 58.16 ± 15.57 *μ*g/g of lead in bone [9].

Many inconsistencies found in the concentration of lead found in the literature are probably related to the fact that whole blood is the most widely used biomarker of exposure. When different animal studies are compared and only whole blood lead is measured and displayed, results will likely not be consistent with the true exposure. As known for quite some time now, lead in bones is the best direct measure related to the degree of exposure to lead [15, 16]. In a study on the effect of lead on rat fetuses [10], significant differences were found in size and body weight between the following groups that were exposed to lead acetate in the mother's drinking water 0.250 mg/L and 500 mg/L. The authors concluded that there was an inverse relationship between the amount of lead to which the fetuses were exposed and the size and weight of the animals, and that lead interferes with normal development since the very early stages, leading to a delay in bone development. It must be mentioned that in the real environment coexposures are certainly not an exception but are many times the case. And some toxicants are bone-seeking agents, such as lead, and could also be easily determined in bone. A case in point is lead and fluoride, both found in industrial areas, and whose coexposures increase by 2-3 times the concentrations of lead in calcified tissues and whole blood, not changing the fluoride concentrations. Interestingly, the enamel defects normally found in rats under conditions of high fluoride exposure are much worsened in the presence of lead [17]. Therefore, the careful measurement of lead in bone is not only very important for the better understanding of the lead amount to which the animal was exposed but is also important to understand possible outcomes such as function of organs and cells.

Since in animals the bone is easily obtained, this study supplies the details of how to obtain accurate measures and supports the need to use bone as the best marker of internal dose.

## 4. Conclusions

The first finding of this study shows a 5 times increase in the concentration of lead in the bone found in the 60-day-old rats in comparison with the 28-day-old rats, with no changes found in the whole blood lead concentrations of these animals. This finding lends additional support to the importance of bone as a tissue that stores lead during the remodeling process characteristic of growth. Lead determinations in bone should be a requirement when growing animals exposed to lead are analyzed. So far, most studies in animals only determine lead in whole blood as a way to characterize the exposure to lead. This study shows differences in lead concentrations in bone and whole blood in animals exposed to lead for 28 and 60 days. Finally the differences in body weight in 60-day animals were also presented.

## Figures and Tables

**Table 1:** Summary of rat studies with lead determinations in whole blood and bone.

Authors	Special condition	Age when study ended (days)	Gender	Treatment groups	Exposure period (days)	Lead concentration in bone (*μ*g/g)	Lead concentration in whole blood (*μ*g/dL)	Main outcome
[8]	Normoxic	111	Female	1000 mg/L	91	632.29 ± 94.23	48.28 ± 8.54	Negative effect on bone growth and bone properties was found in the lead/hypoxia group
	Hypoxic	111	Female	1000 mg/L	91	700.03 ± 78.52	43.78 ± 7.20	

[9]		110	Male	500 mg/L	84	32.23 to 93.43	11.01 to 23.21	Chronic lead exposure causes hippocampus damage

[10]		20 in uterus	Fetuses	250 mg/L 500 mg/L 500 mg/L +Vit. E control	20 (gestation time)	—	—	Rats exposed to lead showed delayed growth and altered ossification Vit. E/Pb worsened condition

[11]		70	Female	250 mg/L 1000 mg/L	21 days of treatment followed by 27 days of exposure to unleaded water	—	21-day group—50 49-day group—93.4	Lead interference with chondrogenesis is more pronounced than with bone formation in growing rats

[12]		—	Female	50 mg/L	126 (~18 weeks)	30.99	9.16	Lead intoxication induces effects similar to osteoporotic diseases

[6]	Pb	81	Female	30 mg/L	84 (~12 weeks)	22.6 ± 8.5	6.8 ± 1.7	Coexposure to lead and fluoride increases between 2 and 3x the amount of lead in calcified tissues, such as dentin, enamel, and bone.
	Pb + fluoride			30 mg/L	84 (~12 weeks)	76.7 ± 11.0	14.2 ± 2.6	

**Table 2:** Lead concentrations found in whole blood of controls (C-28D and C-60D) and animals exposed to lead (30 mg/L) in the drinking water since pregnancy to day 28 (Pb-28D) and day 60 of life (Pb-60D).

	C-28D	C-60D	Pb-28D	Pb-60D
Lead (*μ*g/dL) in whole blood	1.2 (±0.7)	1.6 (±1.5)	8.0 (±1.1)*	7.2 (±0.89)*

*P < 0.001 for differences with age-matched controls.
